# A Versatile Simple Capture Assay for Assessing the Structural Integrity of MHC Multimer Reagents

**DOI:** 10.1371/journal.pone.0137984

**Published:** 2015-09-21

**Authors:** Brendan K. Reed, Laura B. Chopp, Courtney S. Malo, Danielle N. Renner, Virginia S. Van Keulen, Megan A. Girtman, Wendy N. Nevala, Kevin D. Pavelko, Diana Gil, Adam G. Schrum, Aaron J. Johnson, Larry R. Pease

**Affiliations:** 1 Department of Immunology, Mayo Clinic College of Medicine, Mayo Clinic, Rochester, MN, United States of America; 2 Department of Medicine, Division of Hematology, Mayo Clinic College of Medicine, Mayo Clinic, Rochester, MN, United States of America; University of Nebraska-Lincoln, UNITED STATES

## Abstract

Antigen-specific T cell responses can be visualized using MHC:peptide multimers. In cases where robust T cell controls are not readily available to assess the integrity of multimer reagents prior to analyzing limited sample, the ability to assess the structural integrity of MHC multimers before their use in critical experiments would be useful. We present a method to probe the structural integrity of MHC multimers using antibodies specific for conformational determinants. Beads coated with anti-mouse Ig are incubated with conformation-specific mouse monoclonal antibody and then with fluorescently tagged MHC multimer. The ability of the bead to capture the labeled multimer can be measured semi-quantitatively by flow cytometry. In this manner, the correct folding of MHC multimers can be visualized and batches of multimer can be compared for quality control. Because there are multiple conformational epitopes formed by various molecular interactions among heavy chain, peptide, and β_2_M, this capture assay can assess the fidelity of each aspect of multimer structure, depending on the availability of antibodies. The described approach could be particularly useful for studies using irreplaceable samples, including patient samples collected in clinical trials.

## Introduction

MHC multimers including tetramers [1] and pentamers [2] provide a powerful way to visualize antigen specific T cell responses in both experimental and clinical immune assays. The multimers can be acquired from both commercially supported resources and government supported suppliers such as the NIH Tetramer Core Facility, or assembled in the laboratory for measuring immune responses to commonly-studied or novel-hypothetical antigens. The multimer reagents can be expensive, time consuming to acquire, and decay in storage, representing uncontrolled reagents at the time of their use. Often robust positive control T cells may not be available to assess the reagents functionally. This is particularly problematic when using MHC multimers to investigate less well defined T cell responses, such as those characterized by low T cell frequencies or by specificity to hypothetical antigens being evaluated for relevance to cancer, infection, or autoimmune disease. These problems are magnified when studying irreplaceable clinical trial samples where having confidence that reagents are biochemically intact is crucial.

**Fig 1:**
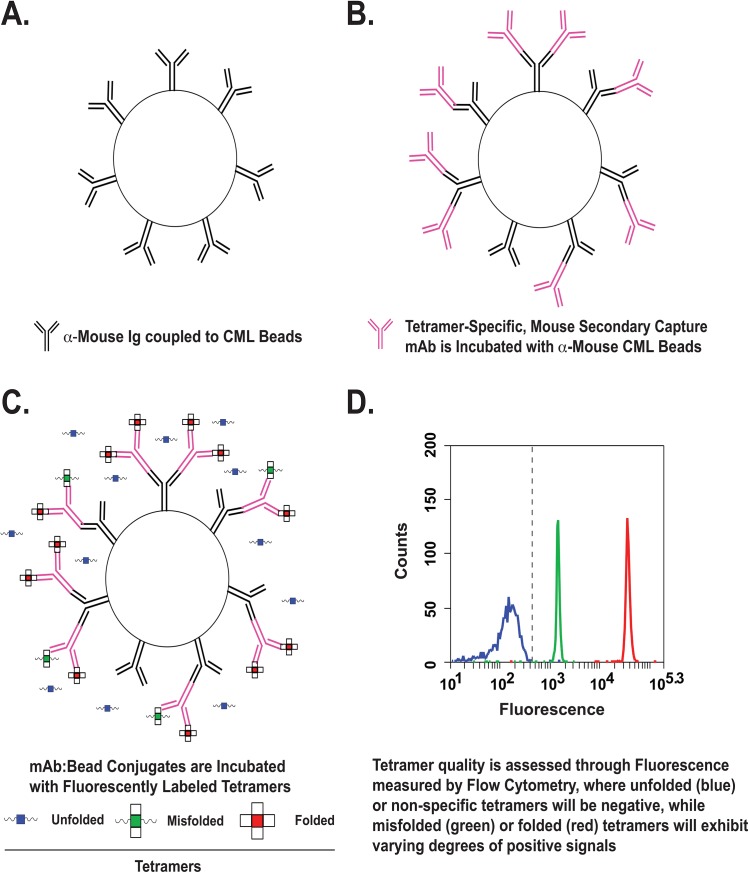
A schematic overview for measuring multimer quality by IP-FCM. (A-B) Polyvalent mouse Ig is covalently linked to CML beads, then incubated with a tetramer specific, mouse secondary capture mAb. (C) These bead:secondary mAb conjugates are incubated with fluorescently labeled multimers to capture fluorescently labeled complexes containing MHC moieties expressing conformational antibody epitopes. (D) The binding of tetramers to antibody coated beads is assessed semi-quantitatively by flow cytometry.

To address this problem, we have devised a versatile, flow cytometry-based, capture assay to probe the structural integrity of MHC multimer reagents. The assay is conceptually illustrated in Fig 1. In principle, the idea is to immobilize on a bead antibody specific for conformational determinants expressed on a properly folded MHC molecule. When a fluorescently tagged multimer is bound to the antibody coated bead, the conjugate becomes labeled and can be visualized by flow cytometry. The amount of multimer bound to bead is quantified by median fluorescence intensity with flow cytometric analysis. Only a small amount of the multimer reagent is required for the assay.

## Materials and Methods

### Antibodies

H-2 specific antibodies [3–6] Y-3, B8-24-3, B22-249.R1, 28-13-3, 28-14-8 (anti-alpha3 identical in H-2D^b^/L^d^), 25D1.16, 22-C5.9, and human HLA specific antibodies [6–15] W6/32, MB40.5, BB7.2, and L368 have been described (Table 1). Donkey anti-mouse Ig polyclonal antibody (DKαMS) was acquired commercially (Jackson ImmunoResearch, West Grove, PA).

**Table 1:** Antibody specificities for the conformational dependent clones utilized to assess the structural integrity of both murine and human multimer reagents.

Antibody Clone Name	Specificity	Reference #
Y-3	H-2K	6
B8-24-3	H-2	7
B22-249.R1	H-2D	7
25D1.16	K^b^:SIINFEKL	8/9
22-C5.9	K^b^:SIINFEKL	8/9
W6/32	HLA-A,B,C	10/11
MB40.5	HLA-A,B,C	10/11
BB7.2	HLA-A,B,C	10/11
L368	hB_2_M	10/11
28-14-8	Alpha3 (H-2D^b^/L^d^)	14/15

### Multimers

Phycoerythrin (PE) conjugated multimers were purchased from Beckman Coulter (Survivin [T20035, S01076], CMV [T20100], Mart-1 [T01008], HLA-A2 negative [T20224]), Medical Biological Laboratories (Her2 [TS916], Muc1 [TS915], HIV-GAG [TS941], or Proimmune (Tyrosinase [JP/2490-09], CMV [KP/3668-03], and FLU [KP/3589-19]) or allophycocyanin (APC) conjugated tetramers assembled experimentally: D^b:^VP2, K^b^:SIINFEKL, K^b^:SIINFEQL, and K^b^:SIYRYYGL using a described procedure [1,12].

### Western blot analysis

1 μl of tetramer reagent was subject to reducing SDS-PAGE (10% acrylamide/bisacrylamide, Bio-Rad) and transferred to nitrocellulose membranes (Bio-Rad) by standard methods. Membranes were blotted with SA-HRP at a concentration of 1:10,000 (Jackson Immunoresearch). ECL substrate (GE Healthcare Life Sciences) was used as a developing reagent. The reaction was visualized using film (GE Healthcare Life Sciences) and quantified using ImageJ software.

### Preparation of beads

A detailed protocol including all recipes, product details, and relevant controls for the preparation of CML-IP beads has been described previously [16,17]. Briefly, IP beads were generated by covalently coupling primary amine groups of donkey polyclonal anti-mouse immunoglobulin (DkαMS) antibody to free carboxyl groups present on CML (carboxylate-modified latex) beads. 1.8 X10^16^ beads were washed 3 times in 2-(N-morpholino)ethanesulfonic acid (MES) buffer and resuspended in 50 ml MES buffer.

20 ml of freshly prepared EDAC (50 mg/ml (1-ethyl-3-(3-dimethylaminopropyl)carbodiimide hydrochloride)-MES buffer was added to activate the carboxyl groups, followed by gentle mixing for 15 minutes, making sure the beads did not settle. The beads were washed 3 times in PBS and resuspended in 50 ml PBS. 50 ml DKaMS (≥0.25 mg/ml) was added to the activated CML beads and then mixed for 3–4 hours on a horizontal shaker. The coupled beads were washed 3x in PBS and resuspended in 100 ml QBS (1 X PBS, 1% bovine serum albumin, 0.02% azide) and stored at 4°C.

### Assay conditions

1 μl CML beads coated with DKαMS Ig were incubated with 0.5μg of a capture antibody (MHC heavy chain-specific, β_2_M-specific, or control Ab) in 50 μl IP-FCM (Immunoprecipitation detected by flow cytometry) buffer (50 ml 1 M Tris−HCl, pH 7.4, 100 mM NaCl, 5% fetal bovine serum, 0.02% sodium azide) for 40 minutes on ice to prepare enough reagent for 8–10 assays. The mixture was washed 2x with 1 ml IP-FCM buffer and then resuspended in 50 μl IP-FCM buffer. CML/capture Ab conjugate was placed into the wells of a 96 well chimney bottom plate. All multimer stocks were kept at a concentration of 0.5mg/mL, where staining was performed at a dilution between 1:500–1:1000 (multimer:IP-FCM buffer) for 40 minutes on ice. The preparations were washed twice with 200 μl of IP-FCM buffer and spun at 3500 rpm for 5 minutes at 4°C. Finally, the preparation was resuspended in 100 μl IP-FCM buffer and transferred to FACS tubes for analysis by flow cytometry.

### Viral Infection/Animal Use

Mice obtained from the Jackson Laboratory (Bar Harbor, ME) and Dr. Chella David’s Immunogenetics mouse colony (Mayo Clinic, Rochester, MN) were injected intracranially with 2*10^4^ PFU of recombinant TMEV. Six days later central nervous system infiltrating lymphocytes were isolated and analyzed by flow cytometry for the presence of virus-specific H-2D^b^:VP2_121–130_ T cells [18,19]. The animals were treated according to Mayo Clinic Institutional Animal Care and Use Committee (accreditation number 000717) guidelines, and the Mayo Clinic IACUC specifically approved this study (Protocol #A57813). The mice were housed five animals per cage and bedding changed weekly or as needed. Mice were fed daily as needed; water was monitored daily and changed at least once a week. Animals were anesthetized briefly with isoflurane (equilibrated vapor in a bell jar with animals monitored continuously and checked for unresponsiveness using a roll test) for intracranial injection, and euthanized for further study prior to development of observable symptoms. Euthanzia was performed with CO_2_ overdose in a two-step process in their home cage using 2.5 L/min CO_2_ for 1 or until animals appear anesthetized, followed by an 11 L/min flow rate for 3 minutes or until respiration, heart beat and reflexes are absent.

## Results

**Fig 2:**
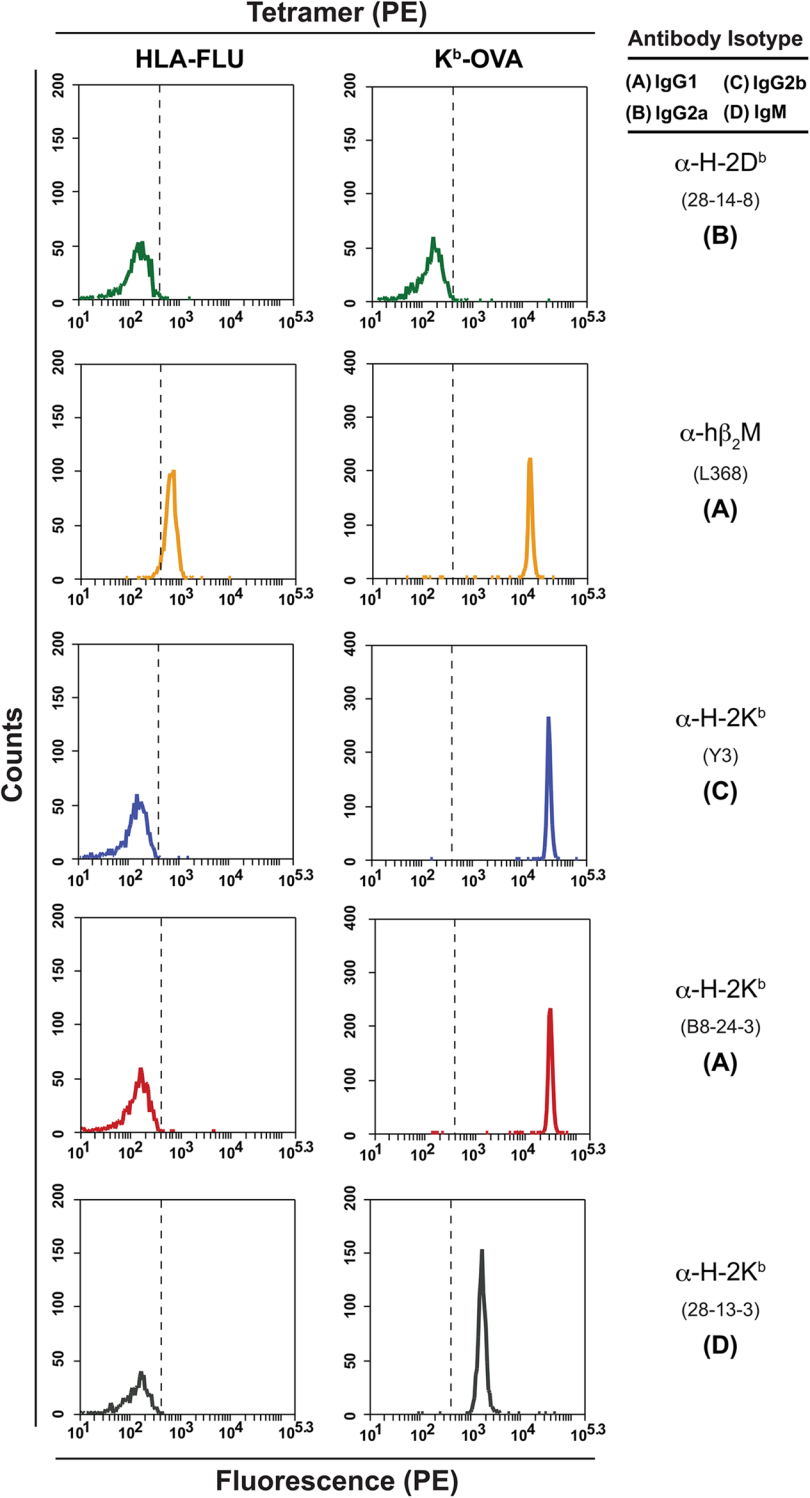
The IP-FCM platform can accommodate multiple antibody isotypes in the secondary capture phase. Antibodies of varying isotype, specific for conformational epitopes on a mouse class I tetramer were used to probe a human and mouse class I tetramer. The mouse tetramer was detected with comparable efficiency using all four isotypes tested.

**Fig 3:**
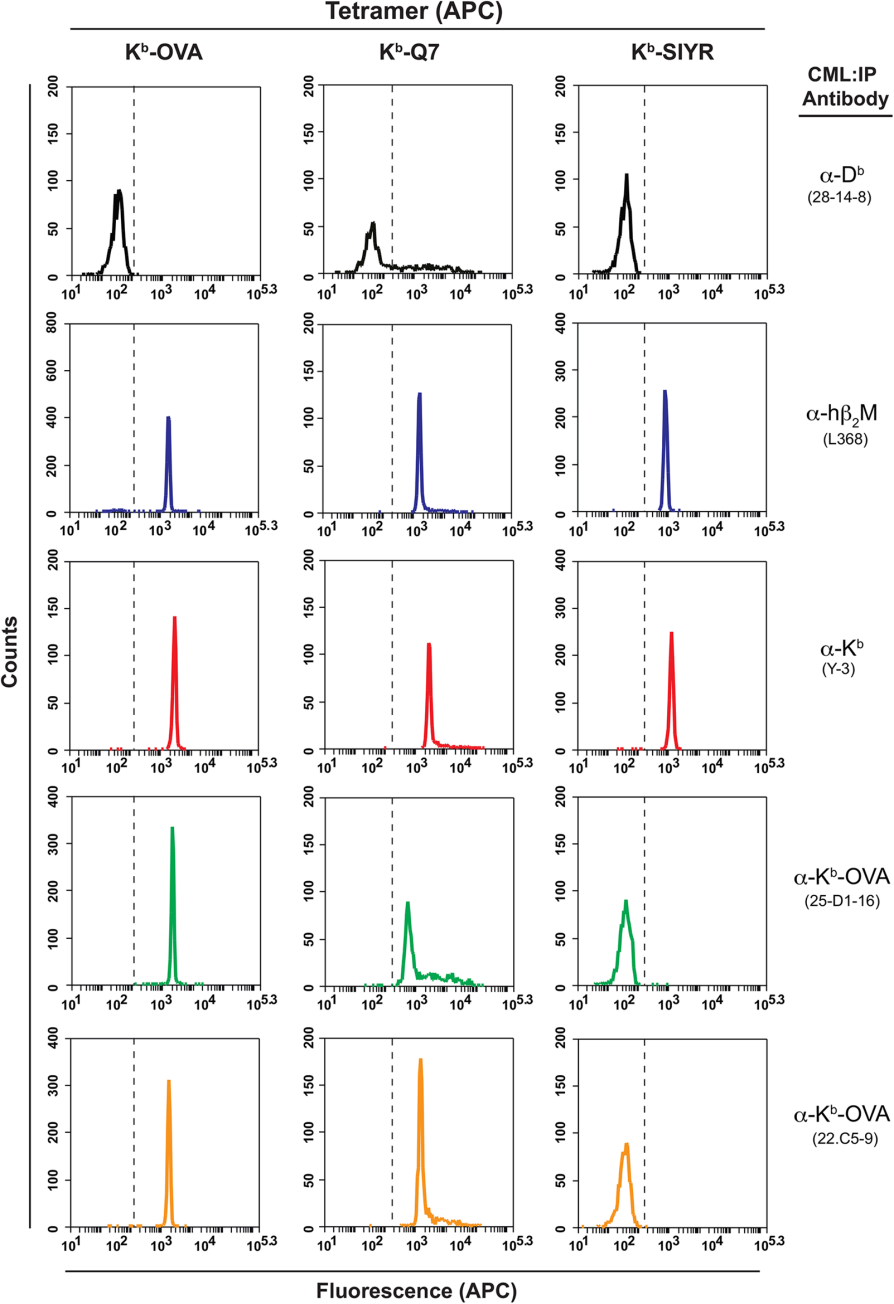
Fine specificity of folded H-2K^b^ tetramers. K^b^ tetramers expressing the peptides SIINFEKL, SIINFE**Q**L, and SIYR were assessed with a panel of negative control (B22-249.R1), cross reactive, anti-hB_2_M (L368), allele-specific, anti-H2-K (Y-3), and peptide-dependent/allele-specific antibodies, anti-K^b^:SIINFEKL (25D1.16 and 22C5.9).

The integrity of the MHC domains of the multimer reagent is indicated in our assay by the ability of bead bound conformation-specific antibody to capture the fluorochrome-labeled multimer to the bead (Fig 1). Because many multimers can be captured by a single bead, and each multimer contains a fixed number of fluorochrome moieties, the assay is semi-quantitative [16,17,20]. Using beads coated with a polyclonal anti mouse Ig antibody proved sensitive and versatile, allowing any selected capture mouse antibody specific for the targeted multimers to be used. The capture antibodies used can be IgM, IgG1, IgG2a or b, and likely any other isotype (Fig 2). Both purified antibody and culture supernatant were effective sources of antibody to assess multimer structures. Antibodies specific for particular MHC alleles (Fig 3), for peptide-MHC quaternary epitope determinants, or to shared epitopes such as the β2-microglobulin (β_2_M)-epitope recognized by Ab L368 (Fig 2) were employed to assess batch to batch variation among tetramer preparations or the integrity of panels of tetramers to be used in T cell assays. Because human β_2_M typically is used to prepare most multimers, due to its higher affinity for both mouse and human class I heavy chain, the Ab L386 (anti- human β_2_M) was used to probe both mouse and human multimers [14].

**Fig 4:**
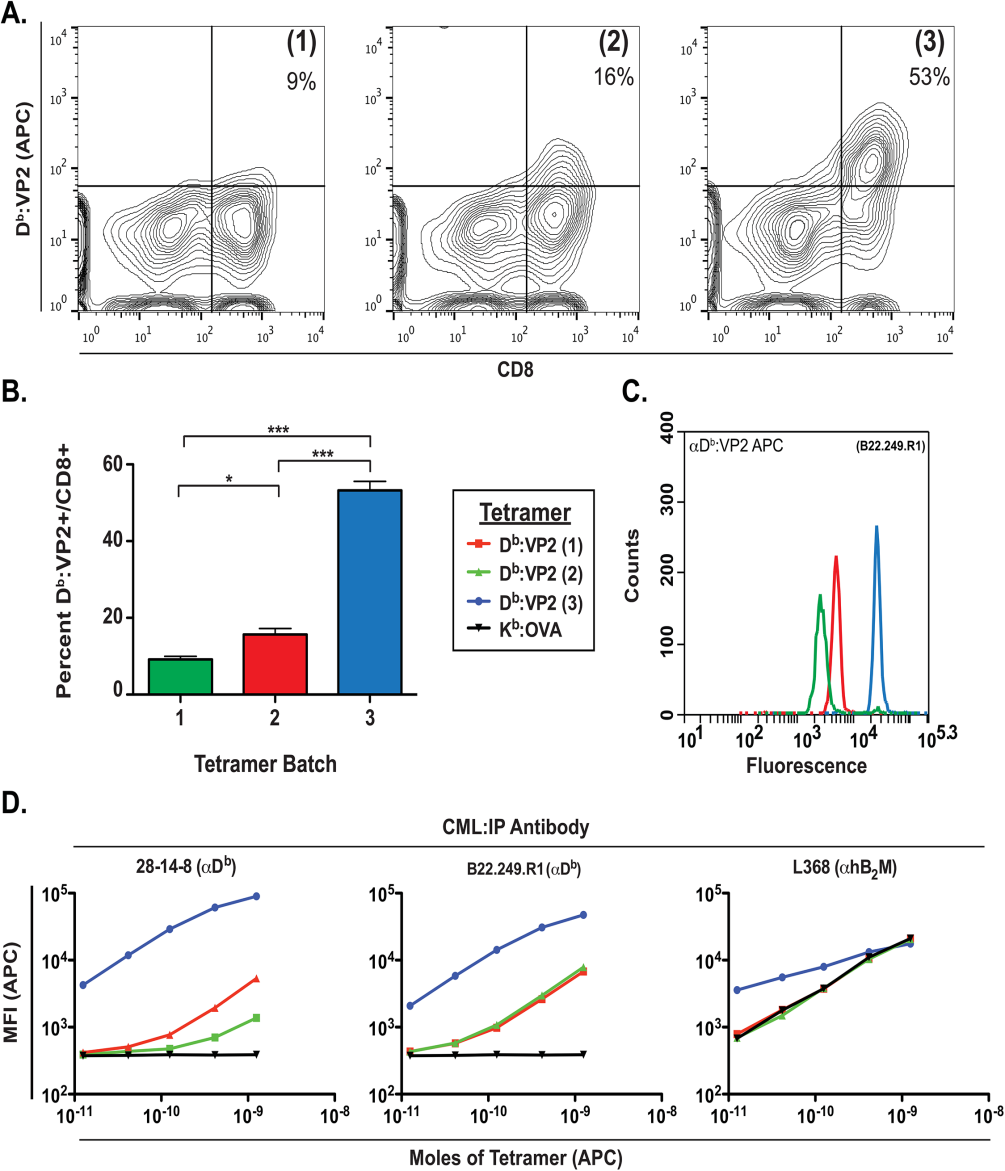
Characterizing functional reactivity of D^b^:VP2 tetramers. Three independently produced batches of D^b^:VP2 tetramers were studied to assess their ability to measure the antigen specific T cell response following TMEV infection. Brain infiltrating leukocytes were isolated from TMEV infected mice as described [12]. (A) Flow cytometric assessment with batch 1, 2, and 3 of D^b^:VP2 tetramers in BILs from TMEV infected mice. (B) Batch differences in detectable percentages of D^b^:VP2/CD8 positive BILs from infected mice. (C) Corresponding fluorescence of D^b^:VP2 tetramers following capture with the D^b^ specific antibody, 28-14-8.

To illustrate how this technique can be applied, we assessed three independently produced batches of a tetramer designed to present the H-2D^b^-restricted immunodominant epitope from Theiler's Murine Encephalomyelitis virus (TMEV), VP2_121-130_ [18]. Mice were infected intracranially with TMEV; seven days later, CD8+ brain infiltrating lymphocytes (BILs) were analyzed for their antigen specificity [18] using the three batches of D^b^:VP2 tetramer (Fig 4). While batch three provided a detection rate of 53% for Db:VP2+/CD8+ BILs in infected mice, batches one and two stained T cells from these same mice with lower efficiencies of 9% and 16% respectively (Fig 4A and 4B). We analyzed these tetramers with our assay and measured their fluorescence intensities when immunoprecipitated with an antibody specific to the D^b^ heavy chain or β_2_M and compared these findings directly to the functional ability to stain antigen specific T cells (Fig 4C). The staining efficiency of these tetramers correlated with the results obtained from our assay, where batches one and two had low fluorescence intensity, while batch three had the highest fluorescence intensity (MFI). While all tetramer stocks were kept at 0.5mg/mL, we performed a titration series where we compared Moles of protein to their corresponding MFI. We observed a dose response in MFI for all three batches of tetramer, with decreased capture in batches one and two relative to batch three, a finding corresponding with their functional staining efficiencies. All three tetramer batches exhibited some folding integrity as shown by capture with L368 antibody specific for human B_2_M (Fig 4D).

**Fig 5:**
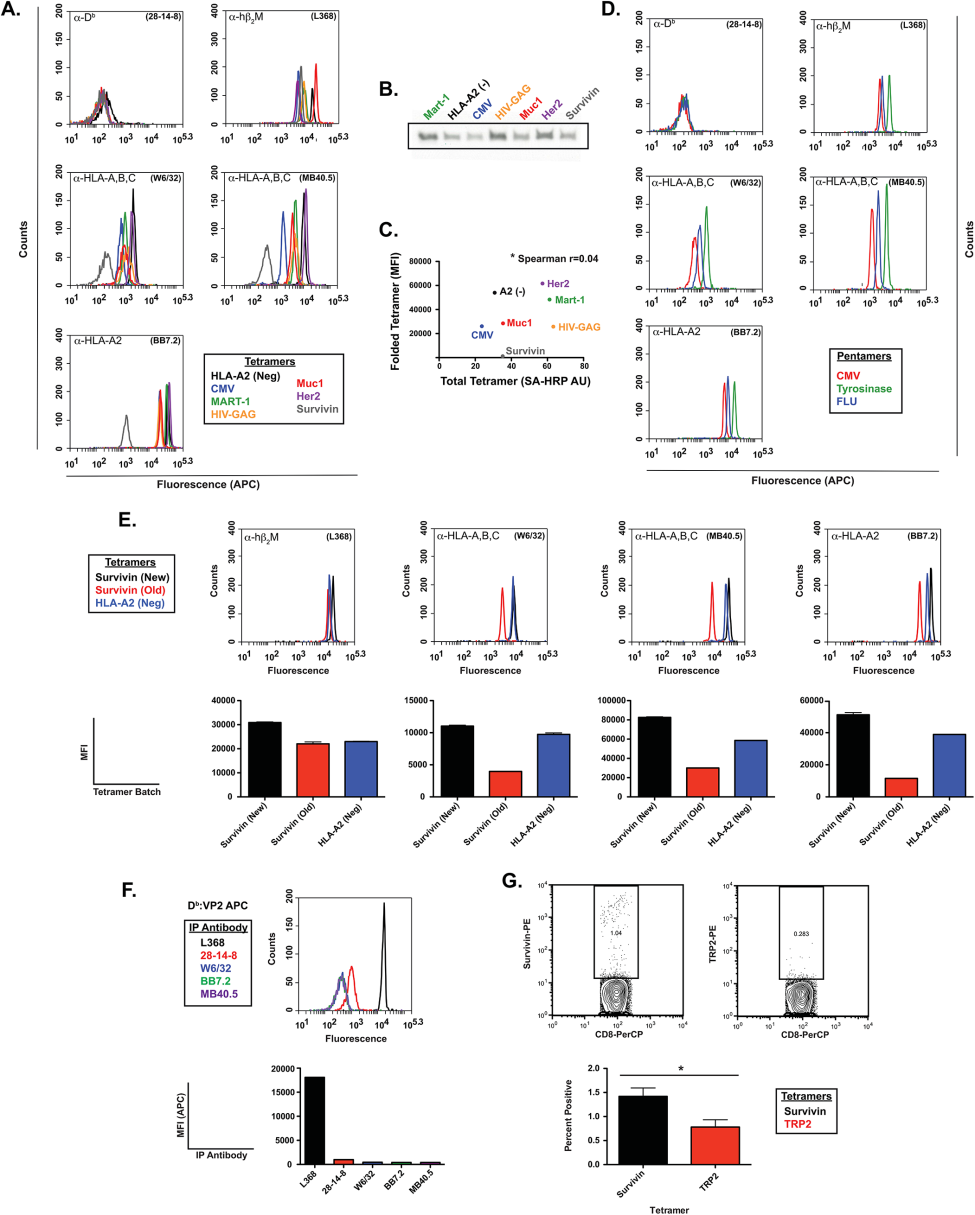
Visualizing variability in human tetramer reagents using IP-FCM. (A) Flow cytometric assessment of commercial batches of human tetramers using antibodies detecting conformational determinants expressed by folded MHC class I molecules. (B) Western blot quantification of monomeric heavy chains present in the tetramer reagents analyzed in panel A. (C) Absence of significant correlation between heavy chain concentration and detection of correctly folded class I molecules in the tetramers measured by IP-FCM. (D) Flow cytometric assessment of commercial batches of human pentamers by IP-FCM. (E) Comparison of two batches of HLA-A2:surviving peptide with a correctly folded commercial standard. Top row, examples of flow cytometry characterization of tetramer confonformation; bottom row, quantification of triplicate estimates with tight error estimates. (F) Negative control mouse heavy-chain tetramer showing absence of reactivity with human-specific heavy chain antibodies. (G) Detection of survivin-specific T cells in HLA-A2 transgenic mice using HLA-A2:surviving tetramer reagent and an irrelevant HLA-A2:TRP2 tetramer control.

We next used our approach to evaluate several batches of HLA tetramers and pentamers acquired commercially to measure antigen specific T cell responses in vaccine clinical trials. The tetramers were probed with a series of antibodies specific for human class I including β_2_M, pan-HLA class I-specific, allele specific antibodies, and compared to signals obtained from a murine specific, functionally active negative control, 28-14-8 (Fig 5). Remarkably, we found wide variation in the structural integrity of these multimers (Fig 5A), even though they are all provided at the same protein concentration (0.5mg/mL), highlighting the importance of using a quality control assay to evaluate multimers, particularly in assays where T cell lines are not readily available to evaluate the reagents functionally. Differences in the specific activity of multimer reagents measured by IP-FCM was not reflected in a parallel analysis of protein concentration where all preparations were found to be approximately equivalent, suggesting that batches of reagent might differ in the degree of correct folding. Western Blot analysis using avidin peroxidase as a probe revealed a range of quantities of biotinylated protein (presumably class I heavy chain by molecular weight) for each tetramer analyzed (Fig 5B). However, corresponding Median Fluorescent Intensity (MFI) testing the same tetramers was non-correlative (Fig 5C); with a Spearman r value of 0.04 (P = 0.964). Similar variability was also detectable when probing HLA pentamers (Fig 5D). These findings indicate that assessing multimer reagents by quantifying the amount of biotin conjugated heavy chain may not be adequate.

Among the human multimers we analyzed was an HLA-A0201:pSurvivin- ELTLGEFLKL tetramer reagent from Beckman Coulter. We noted a poor signal in three of our assays using the heavy chain dependent antibodies W6/32, MD40.5, and BB7.2 suggesting the reagent may not be folded correctly. Aspects of the tetramer structure appear to be present, as an antibody specific for human β_2_M captured labeled avidin, presumably in complex with improperly folded class I heavy chain and β_2_M (Fig 5E). In previous studies we had successfully used this very reagent to detect HLA-A2:Survivin specific T cells elicited in an HLA-A2 transgenic mouse vaccinated intra-cranially with a TMEV virus vaccine encoding the survivin peptide antigen (Fig 5E and 5F). To evaluate the possibility that the tetramer reagent may have decayed upon prolonged storage, we acquired a new batch of the tetramer from the same vendor and compared the old and new batches using our assay. The newly acquired tetramer was captured efficiently by all the antibodies available; whereas the older tetramer reagent was less effectively captured (Fig 5E). These results suggest prolonged storage may alter functional efficacy of multimer reagents.

**Fig 6:**
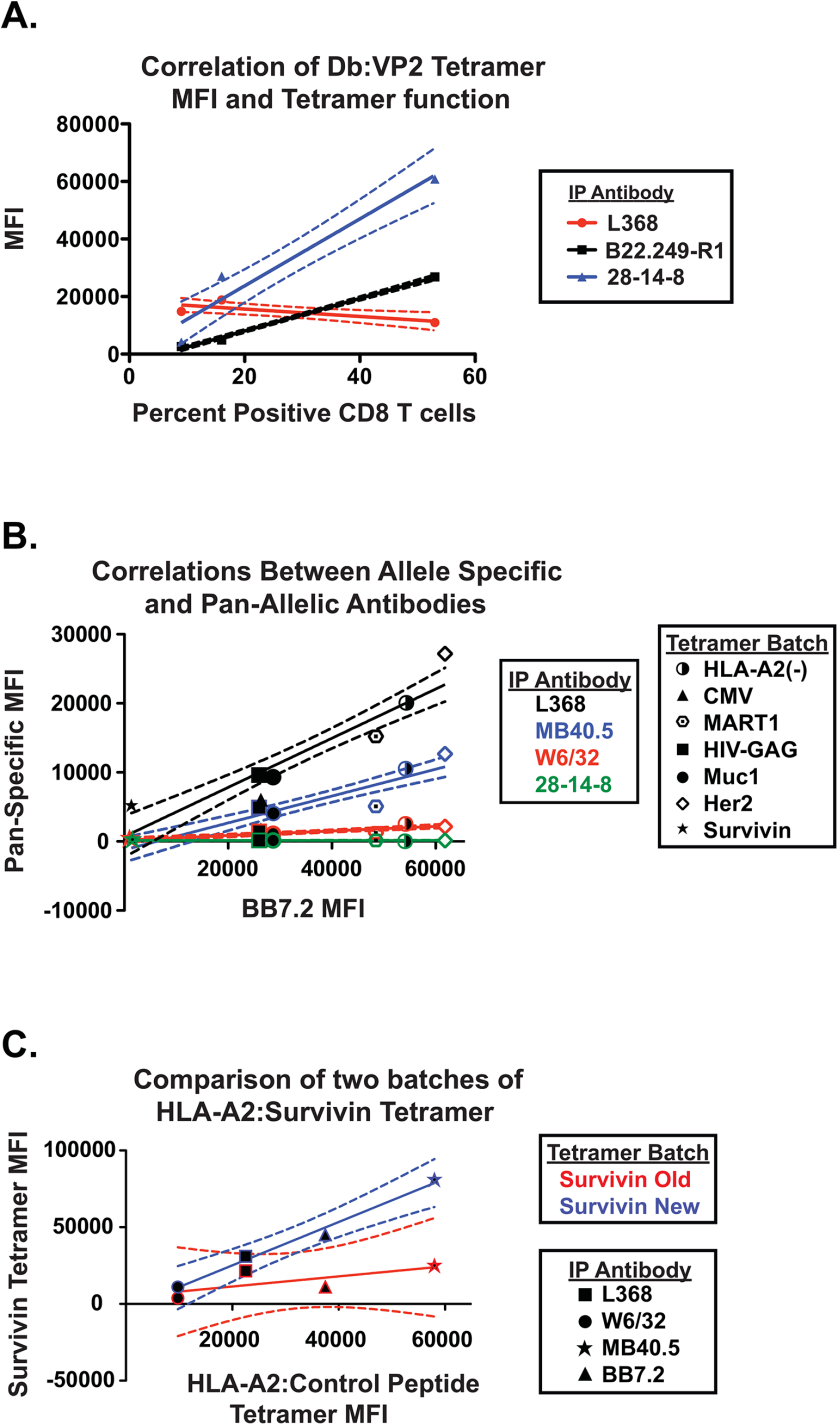
Correlation of structure and function of tetramers capture analysis. (A) Three batches of D^b^:VP2 tetramer were used to assess antigen-specific CD8+ T cells from the same sample of brain infiltrating lymphocytes elicited by infection with intracranial infection with DA strain TMEV. The correlations between the efficacy of detecting antigen specific CD8 cells (from Fig 4) with the MFI using antibodies specific for β_2_M (L368, P = 0.296) or heavy chain (B22.249.R1 and 28-14-8 P = 0.0065) calculated using GraphPad Prism 6 are shown. (B) Seven HLA-A2 tetramers assembled with distinctive peptides were assayed with four different capture antibodies. Highly significant correlations were observed between the abilities of the heavy chain allele-specific antibody, BB7.2, and each of the pan-HLA antibodies, L368, W6/32, and MB40.5, to capture each of the tetramers (P < 0.001 for each case). No correlation was found with mouse H-2 specific antibody in the capture assay. (C) Three tetramers (HLA-A2:irrelevant peptide “-”, HLA-A2:survivin old and new from Fig 5E) were assayed using the four HLA-A2 binding antibodies BB7.2, MB40.5, W6/32, and L368. The positive control tetramer HLA-A2 (-), previously shown to bind robustly to each of the HLA-A2 capture antibodies was (Fig 5A), was used as a standard and compared to the HLA-A2:survivin tetramers. The abilities of the antibodies to capture the HLA-A2 (-) standard was highly correlated with their abilities to capture the new batch of HLA-A2:survivin tetramer (P = 0.0065), whereas there was no significant correlation in the comparison with the old batch (P = 0.296). Comparison of the two survivin tetramers revealed a significant difference in the curves, illustrating that these two reagents are different, despite their similar abilities to bind the β_2_M specific antibody L368.

We also noted that the ability of the antibody specific for β_2_M to capture the tetramer reagents was not always concordant with antibodies specific for conformational epitopes on the class I heavy chain. Whereas the ability of the heavy chain-specific antibodies B22.249.R1 and 28-14-8 to capture tetramers was directly proportional to the ability of the tetramers to bind antigen-specific T cells, no such relationship was observed using β_2_M-specific antibody L368 as the capture reagent (Fig 6A). In our analysis of seven HLA-A2 tetramers, we found a strong correlation between the ability of the heavy-chain allele-specific antibody BB7.2 and the pan-allele specific antibodies W6/32 and MB40.5 and L368 when the tetramers appeared to be properly folded (Fig 6B). However in comparing the ability of these same antibodies to capture the new and old HLA-A2:Survivin tetramers, the relationship held with the new reagent but not for the old reagent, with the largest deviation coming from estimates using L368 as the capture reagent (Fig 6C). We surmise that β_2_M may bind to both folded and misfolded class I heavy chains, and therefore, may provide false positive results when used by itself to interrogate structural integrity of multimer reagents.

## Discussion

Quality control of reagents is a key variable in experimental design. Irrespective of where reagents are acquired, manufactured, or stored, methods to assess their integrity and potency are fundamental to data interpretation and experimental reproducibility. We have devised a simple, versatile assay which can be used to assess the integrity of multimer reagents which are otherwise difficult to assess. The assay takes advantage of the IP-FCM platform [18] and antibody reagents which recognize conformational antibody epitopes formed upon correct folding of complex protein structures. Reagent conformation is an integral property of MHC multimers assembled in vitro from unfolded monomers into complex quaternary structures. The assay can be used to compare batches of multimer preparations to provide assurance that the reagents to be used in experimental and clinical assays contain correctly assembled molecules. Although the assay has been designed and tested for MHC Class I multimers, a similarly compatible platform could be designed for assessing Class II multimers, or generally, any labeled reagent with conformational epitopes and available conformation sensitive antibodies.

The ability to assess folded proteins using this method is dependent on the array of antibodies available. For H-2K^b^, an extensive antibody panel [3,6] allows substantial assessment of structural integrity. We find that spontaneously folded K^b^ molecules express a wide spectrum of conformational epitopes defined on natively folded molecules, indicating high fidelity in molecular structure. This includes determinants generated by single amino acid variations in the bound peptides [15]. In other cases, the available antibody panel is more limited and this new approach is most useful for comparing batch to batch variation in manufacturing and assessing the amount of folded multimer present relative to protein content in an otherwise uncharacterized preparation. In using this assay, consideration should be given to differences in the specific activity of labeling from batch to batch or manufacturer to manufacturer. For example, the number fluorescent tags per avidin will influence the signal detected in the assay limiting the usefulness of this approach to compare folding of reagents prepared using different batches of avidin. However, the relative structural integrity of multimers prepared using the same or similar avidin reagents can be readily assessed using our approach. The availability of an antigen-specific T cell controls to establish the parameters of a functionally folded multimer provide important reference for establishing the limits of the assay. We have used transgenic mice expressing human HLA molecules to generate T cells recognized by human tetramers as a standard, as in the case of survivin. Once the ability of functional multimers to bind to the antigen-specific T cells of interest is characterized, the assay we have established provides a quick and versatile way to monitor the structural integrity of this class of reagents prior to their use to analyze irreplaceable samples or in complex and expensive studies.
